# Multispectral near‐infrared spectroscopy study evaluating the effect of razor design on shaving‐induced erythema

**DOI:** 10.1111/srt.13598

**Published:** 2024-01-26

**Authors:** Chris Boodoo, Dragos Duta, Nathan Swift, Paul Hunter, Anna Khimchenko, General Leung, Karen Cross

**Affiliations:** ^1^ MIMOSA Diagnostics Toronto Ontario Canada; ^2^ Department of Surgery Dalhousie Universitys Halifax Nova Scotia Canada; ^3^ Innovators in Residence Program Nova Scotia Health Authority Halifax Nova Scotia Canada

**Keywords:** dermatology, inflammation, men's grooming, multispectral NIR, non‐invasive imaging, razor design, razors, shaving, skin irritation

## Abstract

**Background:**

While shaving‐induced erythema is a common inflammatory skin issue, there is a lack of quantitative information on how well a shaving product performs in this regard. In this study, multispectral near‐infrared spectroscopy (NIRS) imaging was used to quantitatively and qualitatively measure the extent of shaving‐induced erythema. The research compares a safety razor and a cartridge razor to evaluate their impact on skin irritation.

**Materials and methods:**

Fifty‐nine healthy male volunteers without pre‐existing skin conditions were enrolled. Basic demographics were recorded, and participants' faces or necks were imaged before shaving. Shaving was conducted on the right side of the face/neck with the safety razor and on the left side of the face/neck using the 3‐blade cartridge razor. Images were captured immediately after shaving, at 5 and 10 min post‐shaving.

**Results:**

Tissue oxygen saturation (StO2) measurements demonstrated that the safety razor induced significantly less erythema than the cartridge razor. Immediately after shaving, 40.3% of skin shaved with the safety razor had erythema compared to 57.6% for the cartridge razor. At 5 min post‐shaving, 36.5% of skin shaved with the safety razor had erythema, compared to 53.8% of cartridge razor.

**Conclusions:**

Multispectral NIRS revealed significant differences in shaving‐induced erythema between safety and cartridge razors. Safety razors demonstrated a lower incidence of erythema, suggesting a potential advantage for individuals prone to skin irritation. This study contributes valuable insights into skin irritation and highlights the potential of multispectral NIRS in dermatology research.

## 1 INTRODUCTION

Male grooming has evolved significantly over time. Nowadays, the market is saturated with an extensive array of shaving products, ranging from traditional disposable razors to cutting‐edge electric devices. Despite the remarkable scientific advancements that have transformed this industry, there is a lack of scientific information on how well a shaving product performs as the research and development of such products remains largely unpublished.

Suboptimal shaving may trigger vasodilation in the small blood vessels within the dermis, resulting in hyperemia and erythema.^1^, ^2^ This condition is known as “razor burn”, or shaving‐induced erythema. This can, in turn, provoke a significant inflammatory response in the skin. Moreover, the excessive removal of immature corneocytes from the stratum corneum can impair the skin barrier, inciting inflammation and hyperproliferation of keratinocytes.^3^ Facial erythema can be lasting for months, influencing the quality of life, self‐confidence, and self‐esteem of those experiencing it.^4^

While there are devices that can be used to detect and quantify erythema (e.g., chromameter, high‐frequency ultrasound, and laser speckle contrast imaging), commonly used erythema assessment tools lack objectivity.^5^ For example, traditional examinations of the effects of shaving on skin are based on consumer self‐assessment, typically performed immediately following the shaving process.^1^ In the development of shaving products there is a need for quantification to assess product performance effectively.

The study employs multispectral near‐infrared spectroscopy (NIRS), a non‐invasive technique to create images of tissue layers and identify changes in tissue composition, oxygenation, and blood flow.^6^, ^7^ The aim of the study was to compare the amount of shaving‐induced erythema caused by safety razors and cartridge razors, providing insights into product performance. This research aims to improve the understanding of the impact of shaving on the skin and enhance the development of safer shaving products.

## 2 MATERIALS AND METHODS

### 2.1 Study design

This observational study aimed to assess and compare the extent of shaving‐induced erythema among participants at three time points: immediately after shaving, 5 min post‐shaving, and 10 min post‐shaving.

### 2.2 Study participants

The study recruited 59 healthy male volunteers who provided informed consent and did not have pre‐existing skin conditions in the relevant area. The participants had a mean age of 39.1 ± 13.3 years.

Inclusion Criteria:

- individuals aged 18 years or older who were proficient in English.

Exclusion Criteria:

- presence of any skin conditions in the relevant area that could result in erythema (e.g., skin redness).

### 2.3 Study materials

**FIGURE 1:**
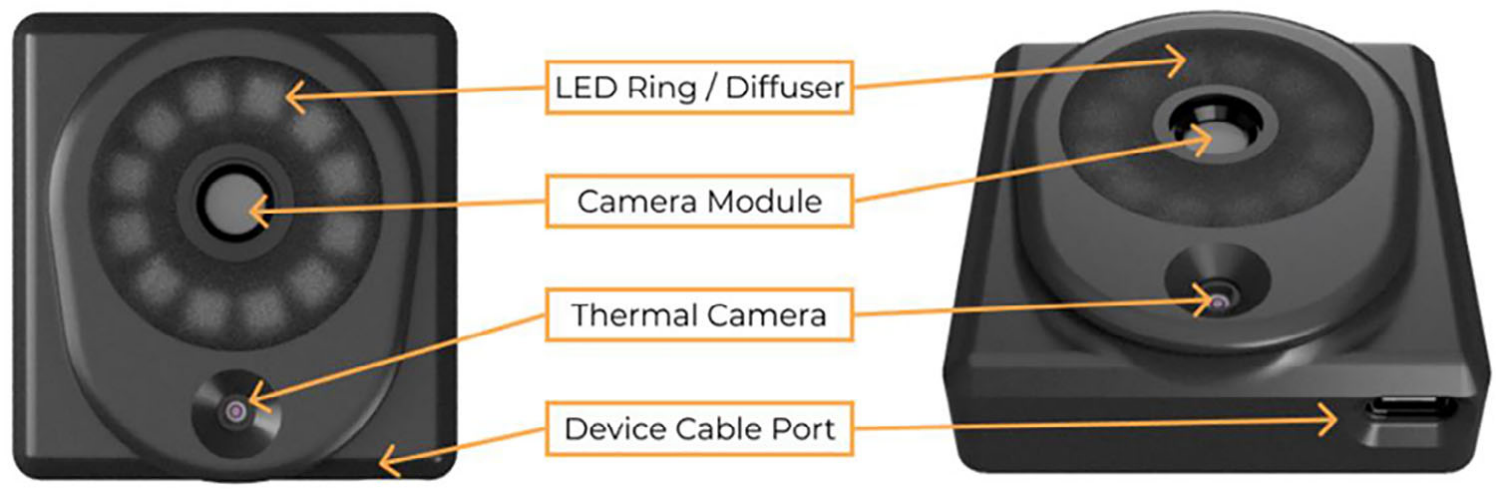
Major components of the Mimosa Pro device.

**FIGURE 2:**
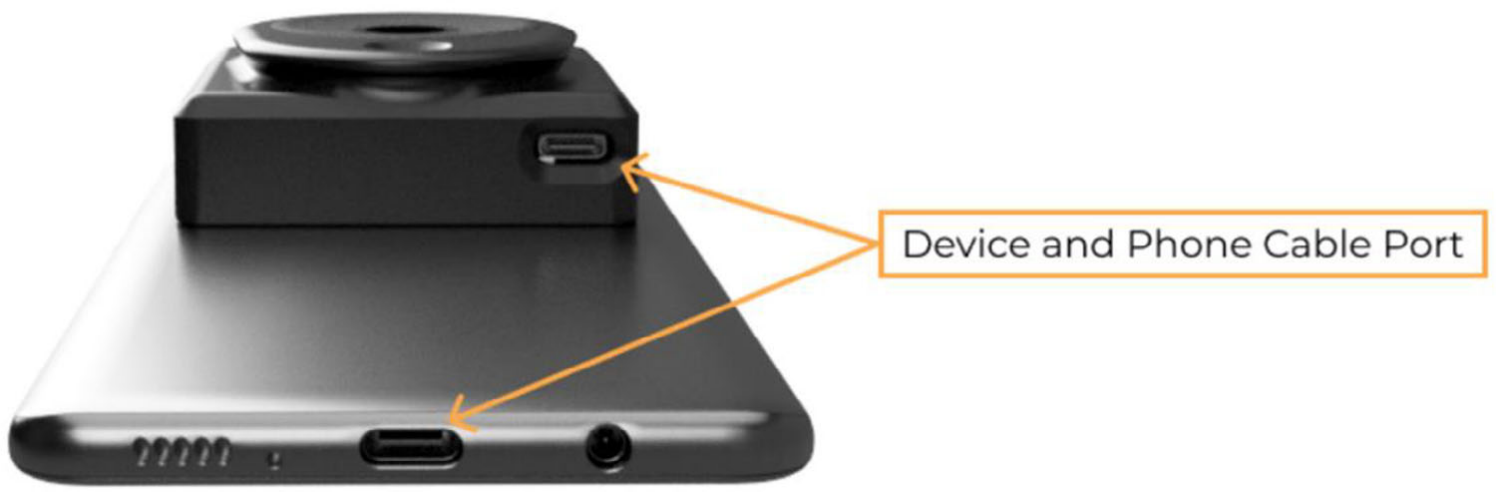
Device and smartphone connection ports.

For the detection of shaving‐induced erythema, multispectral NIRS spectroscopy was used. The imaging device employed in this study is the MIMOSA Pro, developed by MIMOSA Diagnostics Inc. (Figure 1 and 2). This device incorporates a specialized camera that attaches to an accompanying smartphone. It utilizes 12 near‐infrared LEDs to illuminate the tissue with various wavelengths of light, spanning the visible and near‐infrared spectrum (400–1000 nm). The LEDs emit light at discrete wavelengths, and the images are captured in less than 1 s.

Two shaving razors commercially available and marketed in Canada were compared in this study. The first razor, Razor A, was a safety razor (Henson AL13, Henson Shaving, Kitchener, Ontario, Canada). The second razor, Razor B, was a disposable 3‐blade cartridge razor (Xtreme 3 Sensitive Disposable Razor, Schick, North Bergen, NJ, USA). Participants were also provided with shaving foam and were allowed to use it if preferred (Proraso Shaving Foam, Proraso, Florence, Italy).

### 2.4 Study procedures

All consenting participants were initially assessed for basic demographics including age, gender and Fitzpatrick Skin Phototype. Based on the participants decision of shaving either their face or neck, both sides of the participants faces or neck were imaged prior to shaving. Participants were then asked to shave the right side of the area of interest (face or neck) using the safety razor and shave the same area on the left side of their face or neck using the cartridge razor. Immediately after shaving, 5 min after shaving and at 10 min after shaving, both sides of the participants face or neck were imaged.

### 2.5 Data analysis

Shaving‐induced erythema was defined as increase in tissue oximetry (oxygen saturation (StO_2_)) from baseline, measured by multispectral NIRS imaging. StO_2_ is a percent measure of oxygen saturation of a volume of tissue between 0% and 100%. If no increase in StO_2_ was observed (StO_2_ increased less than 10%), then it was defined that the participant did not experience shaving‐induced erythema. The analysis calculated the proportion of the population that experienced shaving‐induced erythema per razor per time point. *t*‐Tests were applied to detect whether there were significant differences between the razors per time point. The Student's *t*‐test for paired samples was used to compare baseline (before shaving) parameters. Statistical significance was considered when the value of *p* < 0.05.

Relative shaving‐induced erythema was another endpoint explored in this study. A relative measure of StO_2_ was calculated per time point for the regions of interest.

The data analysis for this study was conducted using MATLAB version 2023b (The MathWorks, Natick, MA, USA).

### 2.6 Ethics

The research was carried out at the Henson Shaving office in Kitchener, Ontario, and received approval from Veritas IRB (2023‐3170). The nature of the study was explained to all the participants, who agreed to participate by verbal and written consent. All measurements were non‐invasive and participant data was kept confidential.

## 3 RESULTS

**FIGURE 3:**
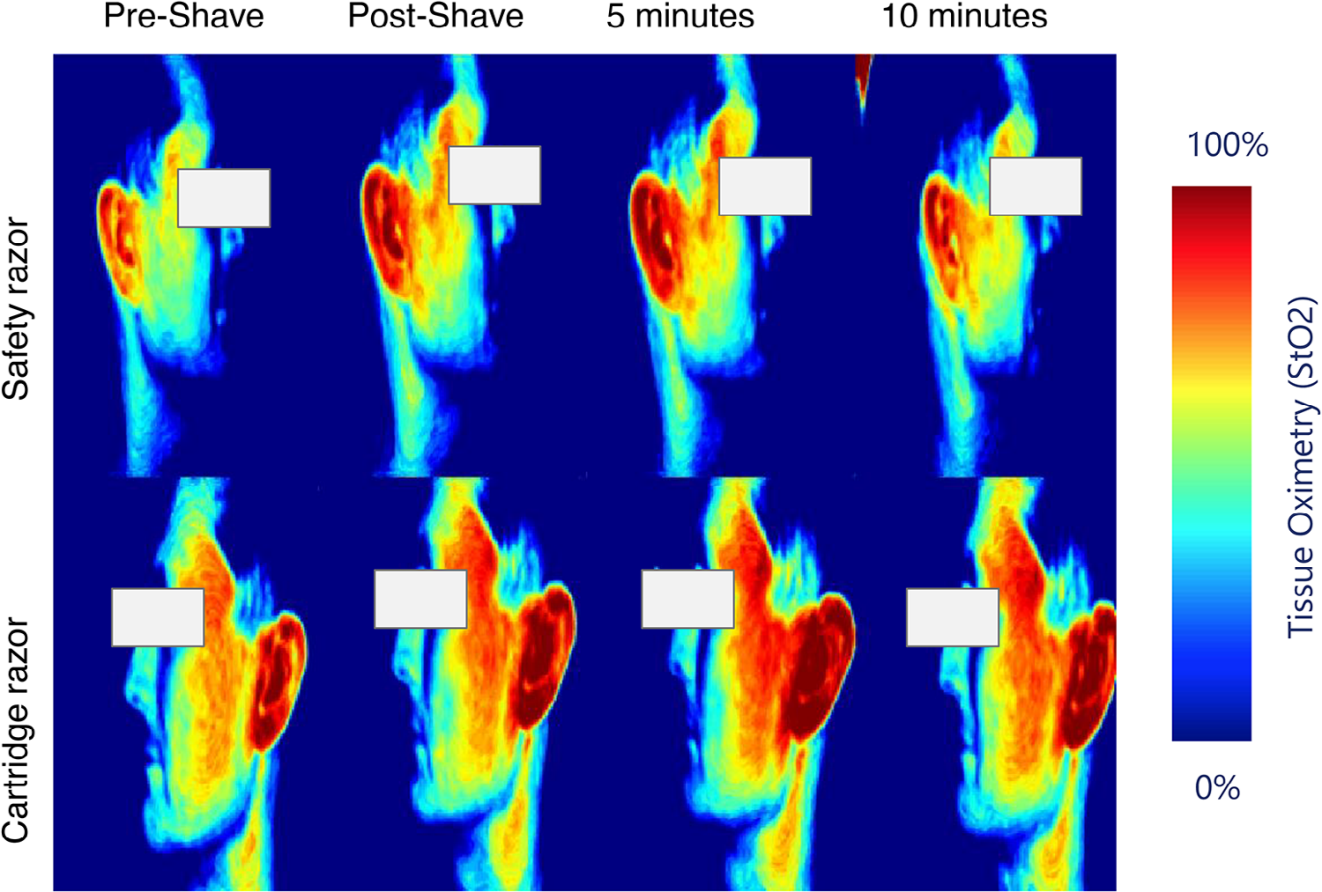
StO_2_ maps of the face of an individual immediately after shaving (Post‐Shave), 5 min after shaving and 10 min after shaving.

To investigate shaving‐induced erythema, StO_2_ via NIRS was measured. Visual insights into the findings are presented in Figure 3, showcasing StO_2_ maps of an individual's face immediately after shaving, 5 min after shaving and 10 min after shaving. Upon close examination of StO_2_ post‐shaving, an evident rise in oxygen saturation is observed for both razors. This participant can be classified as a ‘responder,’ indicating the presence of erythema, as evidenced by a notable increase in oxygenation post‐shaving. Participants exhibiting no significant increase in oxygenation post‐shaving were categorized as “non‐responders.”

The findings regarding the incidence of post‐shaving erythema within the study population are outlined in Table 1. Following the shaving procedure, 40.3% (*p* value < 0.05) of participants exhibited erythema on the side treated with a safety razor immediately afterward. This percentage was notably lower than the corresponding number for the side treated with a cartridge razor, where 57.6% of study participants experienced erythema.

**TABLE 1:** Percentage of population experiencing erythema post‐intervention.

	Safety razor	Cartridge razor
Immediately after shaving	40.3%	57.6%
5 min after shaving	36.5%	53.8%

**FIGURE 4:**
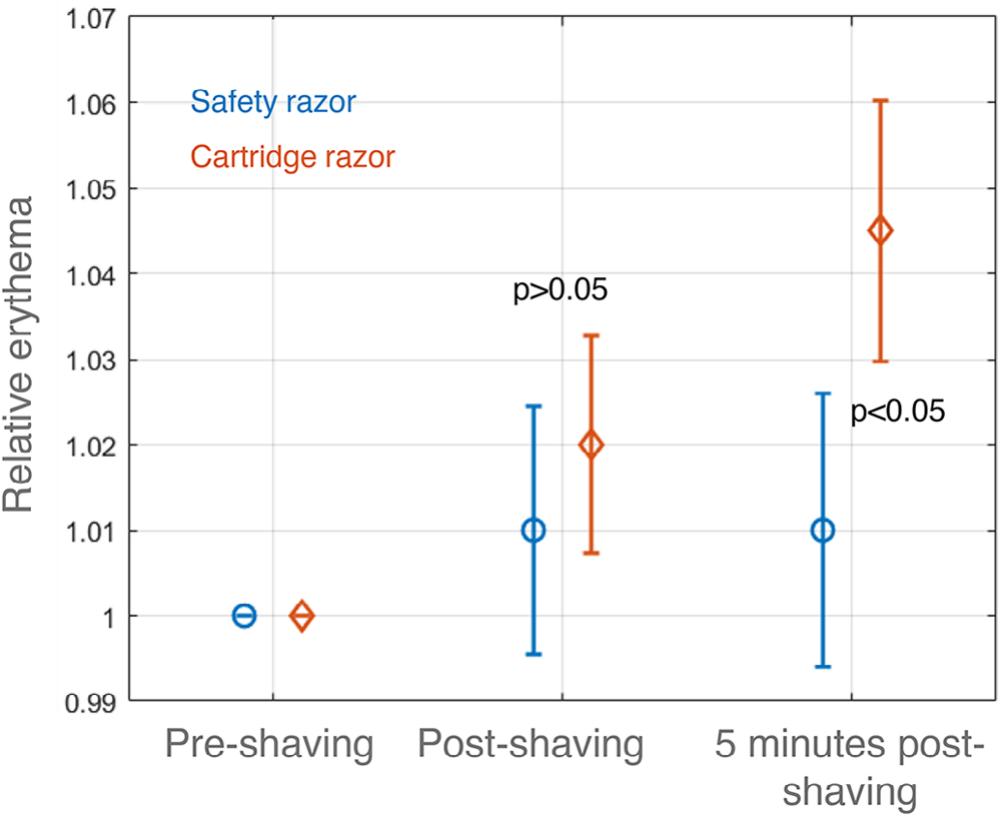
Relative erythema over time for each razor.

The findings related to relative shaving‐induced erythema are illustrated in Figure 4. Upon examination, it was observed that the relative erythema 5 min after shaving with a safety razor was significantly lower (*p* < 0.05) compared to a cartridge razor.

## 4 DISCUSSION

Although a few studies have reported the effects of shaving on skin, there remains limited quantitative data of the extent of shaving‐induced erythema linked to various shaving razor designs. This research has contributed novel insights by employing multispectral NIRS imaging to examine the impact of safety razors versus cartridge razors on shaving‐induced erythema. The results demonstrated a significant difference in the incidence of post‐shaving erythema between the two razor types. Users of safety razors experienced erythema less frequently, consistently displaying lower relative erythema compared to those using cartridge razors.

Understanding of shaving‐induced skin irritation remains a persistent challenge.^3^ The precise origins of this irritation, whether stemming from superficial skin trauma or interactions with the hair shaft signaling to the perifollicular skin compartment, remain largely questionable.^2^ Previous research proposes that shaving irritation may involve the removal of irregular skin elevations, particularly around follicular openings, by the razor blade.^8^ Facial erythema emerges as a result of the dilation of blood vessels in the skin, leading to an increase in blood flow. While transient facial erythema is a normal reaction to emotions, exercise, or exposure to heat, shaving‐induced erythema is specifically triggered by inflammation,^4^ vasodilation, and changes in vascular structure which result in the abnormal increase of hemoglobin in the papillary dermis.^5^

In this study, NIRS was used to detect shaving‐induced erythema, providing insights into the potential of oxygen saturation as a marker for shaving‐induced erythema. Oxygen saturation is a ratio between oxyhemoglobin and deoxyhemoglobin. NIRS technology has the potential to revolutionize the way that skin irritation is studied and evaluated. For example, multispectral NIRS could be used to assess the impact of different shaving products, such as creams and gels, on skin irritation. Additionally, this technology could be used to monitor the effectiveness of interventions aimed at reducing skin irritation, such as the use of moisturizers or other topical agents.

The results highlighted varying levels of erythema induced by safety and cartridge razors. A comparison of oxygen saturation levels immediately post‐shaving and at the 5‐min mark revealed variations in the vascular response. Shaving, as evidenced by the release of neurotransmitters and pro‐inflammatory cytokines, can initiate an inflammatory response in the skin, leading to the dilation of small blood vessels within the dermal layer, potentially resulting in hyperemia and erythema .^9^ While no significant differences were observed in the immediate post‐shaving response, the delayed onset of erythema suggests a potential association with mechanisms related to shaving‐induced skin irritation, microtrauma, or the occurrence of nicks and cuts during shaving.

Shaving presents a unique challenge, requiring the delicate balance of efficient hair removal while minimizing skin injuries. The complexity of shaving is further compounded by the anatomy of male facial hair (e.g., diversity in thickness, length, stiffness, and density, along with an oblique angle of growth) and skin (e.g., heterogeneous morphology, roughness, slower healing properties, and susceptibility to hyperpigmentation).^2^ Shaving razors must delicately manage forces applied to skin and hair, necessitating thoughtful blade design.

The heightened levels of erythema observed in cartridge razors, compared to safety razors, might be attributed to their design featuring multiple blades, leading to increased friction and pressure during multiple passes over the skin.^1^ In contrast, safety razors, with a single blade that glides more gently, reduce the potential for friction and pressure, thus lowering the likelihood of erythema.^10^, ^11^ Refinements in the safety razor design used in this study further contributed to minimizing skin irritation by precisely controlling and limiting razor chatter.

The observed differences in erythema between these two razor types underscore potential advantages for users, particularly those prone to skin irritation. The lower incidence of erythema associated with a safety razor suggests a tangible benefit for individuals seeking a gentler shaving experience.

It is essential to acknowledge the limitations of the study, which may impact the generalizability of the findings. First, individual grooming habits, such as pressure applied during shaving or frequency of razor use, were not controlled for, potentially influencing post‐shaving erythema. Furthermore, the inclusion of shaving foam, a commonly used product in real‐world scenarios, introduces an additional layer of variability to the results. Individual responses to the specific ingredients in the shaving foam, such as eucalyptus oil and menthol, may influence the level of irritation or contribute to its reduction and alleviation of redness. Future research should consider addressing these variables to provide a more comprehensive understanding of the complexities associated with shaving‐induced skin irritation.

## 5 CONCLUSION

The study has showcased multispectral NIRS imaging as a valuable tool for assessing and quantifying shaving‐induced erythema and revealed significant differences in shaving‐induced erythema between safety and cartridge razors. The lower incidence of erythema associated with safety razors suggests a potential advantage for individuals prone to skin irritation. However, it is important to note that the complex mechanisms and individual factors contributing to shaving‐induced erythema warrant further investigation.

## AUTHOR CONTRIBUTIONS

Chris Boodoo: Project administration, Research design, Execution, Original draft, Review & Editing, Approval of final manuscript. Dragos Duta: Execution of research, Review & Editing, Approval of final manuscript. Nathan Swift: Execution of research, Review & Editing, Approval of final manuscript. Paul Hunter: Execution of research, Review & Editing, Approval of final manuscript. Anna Khimchenko: Final draft, Review & Editing, Approval of final manuscript. General Leung: Funding acquisition, Conceptualization, Research design, Methodology, Formal data analysis, Review & Editing, Approval of final manuscript. Karen Cross: Data curation, Review & Editing, Approval of final manuscript. All authors (Chris Boodoo, Dragos Duta, Nathan Swift, Paul Hunter, Anna Khimchenko, General Leung, Karen Cross) have read and approved the final manuscript.

## CONFLICT OF INTEREST STATEMENT

The authors declare no conflicts of interest.

## ETHICAL STATEMENT

The research was carried out at the Henson Shaving office in Kitchener, Ontario, and received approval from Veritas IRB (2023‐3170). The nature of the study was explained to all the participants, who agreed to participate by verbal and written consent. All measurements were non‐invasive and participant data was kept confidential.

## Data Availability

The data that support the findings of this study are available from the corresponding author upon reasonable request.
