# Correction to “Multispectral Near‐Infrared Spectroscopy Study Evaluating the Effect of Razor Design on Shaving‐Induced Erythema”

**DOI:** 10.1111/srt.70314

**Published:** 2025-12-21

C. Boodoo, D. Duta, N. Swift, P. Hunter, A. Khimchenko, G. Leung, and K. Cross, “Multispectral Near‐Infrared Spectroscopy Study Evaluating the Effect of Razor Design on Shaving‐Induced Erythema,” *Skin Research and Technology* 30, no. 2 (2024): e13598, https://doi.org/10.1111/srt.13598.

In the Conflict of Interest section, the text “The authors declare no conflicts of interest” was incorrect. This should have read: “All authors were affiliated with MIMOSA Diagnostics, the manufacturer of the device employed in the study (MIMOSA Pro), at the time of the study.”

The authors apologize for this error and for the inconvenience it may have caused.

